# Deployment of Rotavirus Vaccine in Western Kenya Coincides with a Reduction in All-Cause Child Mortality: A Retrospective Cohort Study

**DOI:** 10.3390/vaccines11081299

**Published:** 2023-07-29

**Authors:** Peter Sifuna, Andrea V. Shaw, Tina Lucas, Bernards Ogutu, Walter Otieno, David A. Larsen

**Affiliations:** 1 Kenya Medical Research Institute (KEMRI), Kisumu 40100, Kenya; peter.sifuna@usamru-k.org (P.S.); lucas.tina@usamru-k.org (T.L.); ogutu6@gmail.com (B.O.); walter.otieno@usamru-k.org (W.O.); 2 US Army Medical Research Directorate–Africa (USAMRD-A), Kisumu 00200, Kenya; 3 Institute for Global Health and Translational Science, SUNY Upstate Medical University, Syracuse, NY 13210, USA; shawan@upstate.edu; 4 Department of Public Health, Syracuse University, Syracuse, NY 13244, USA

**Keywords:** rotavirus vaccine, mortality, morbidity, children, health and demographic surveillance systems

## Abstract

Rotavirus is an important cause of fatal pediatric diarrhea worldwide. Many national immunization programs began adding rotavirus vaccine following a 2009 World Health Organization recommendation. Kenya added rotavirus vaccine to their immunization program at the end of 2014. From a cohort of 38,463 children in the Kisumu health and demographic surveillance site in western Kenya, we assessed how the implementation of the rotavirus vaccine affected mortality in children under 3 years of age. Following its introduction in late 2014, the span of rotavirus vaccine coverage for children increased to 75% by 2017. Receiving the rotavirus vaccine was associated with a 44% reduction in all-cause child mortality (95% confidence interval = 28–68%, *p* < 0.0001), but not diarrhea-specific mortality (*p* = 0.401). All-cause child mortality declined 2% per month following the implementation of the rotavirus vaccine (*p* = 0.002) among both vaccinated and unvaccinated children, but diarrhea-specific mortality was not associated with the implementation of the rotavirus vaccine independent of individual vaccine status (*p* = 0.125). The incidence of acute diarrhea decreased over the study period, and the introduction of the rotavirus vaccine was not associated with population-wide trends (*p* = 0.452). The receipt of the rotavirus vaccine was associated with a 34% reduction in the incidence of diarrhea (95% confidence interval = 24–43% reduction). These results suggest that rotavirus vaccine may have had an impact on all-cause child mortality. The analyses of diarrhea-specific mortality were limited by relatively few deaths (n = 57), as others have found a strong reduction in diarrhea-specific mortality. Selection bias may have played a part in these results—children receiving rotavirus vaccine were more likely to be fully immunized than children not receiving the rotavirus vaccine.

## 1. Introduction

Rotavirus was estimated to be responsible for 40% of all diarrhea-specific mortality in young children prior to the introduction of the rotavirus vaccine (RV) [1,2]. The World Health Organization (WHO) recommended RV to national immunization programs beginning in 2009, with special focus on countries with a high diarrhea-related mortality burden [3]. By the end of 2014, Kenya was among the >70 countries that had introduced rotavirus vaccine into their routine immunization programs [4]. The monovalent (RV1) vaccine, Rotarix, used in Kenya, is orally administered to young infants via a two-dose schedule. As of 2018, RV had been incorporated into the national immunization programs of over 95 countries worldwide [5]. In sub-Saharan Africa, where the potential impact of RV is considerable owing to the high rates of rotavirus-associated mortality, the vaccine has now been introduced in 34 of 47 countries in the African region of the WHO [1]. Several studies around the world have documented the impact of RV on rotavirus-associated hospitalizations and deaths in low- to high-income countries [6,7,8,9,10,11,12,13]. Data for these studies generally utilize national vital registration systems, resulting in incomplete datasets in most low- and middle-income countries (LMICs) like Kenya, where these systems are often insufficient. Additionally, the population-level impact of RV is difficult to estimate in facility-based studies and can best be estimated in population-based surveillance platforms, such as the Kombewa Clinical Research Center’s Health and Demographic Surveillance System (Kombewa HDSS) [14]. Various studies have assessed RV impact on diarrheal illness and diarrhea-specific mortality at the population level in sub-Saharan Africa [9,15,16,17,18,19]; however, the impact of RV on all-cause mortality (indirect benefit) has not yet been determined.

In the absence of a reliable vital registration system, geographically unique demographic surveillance systems such as the Kombewa HDSS can help fill the information gaps and monitor the impact of various public health interventions [20]. As RV is implemented within the Kenyan immunization program, it is imperative to evaluate its impact and effectiveness to inform its future use and to allow for more accurate estimates of the impact of current vaccination strategies on the burden of diarrheal illness among young children [21,22]. Our objective was to examine the impact of the deployment of the rotavirus vaccine among children younger than 3 years using data from a prospective, longitudinal, and population-based surveillance platform in rural western Kenya.

## 2. Materials and Methods

### 2.1. Study Design

We utilized data from the Kombewa HDSS in the rural western portion of Kisumu County, Kenya to create a retrospective cohort of children < 3 years of age from 2012 to 2017. We then tested the impact of the deployment of the rotavirus vaccine in 2014 using an interrupted time series approach. 

### 2.2. Setting

The Kombewa HDSS in Kisumu, Kenya was established in 2007 as a joint operation between the Kenya Medical Research Institute (KEMRI) and the United States Army Medical Research Directorate–Africa (USAMRD-A). The site tracks the demographics of over 160,000 residents. Births, deaths, migrations, immunization status of children under the ages of five and the incidence of various illnesses among other details are routinely captured during each round of updates. Details of the Kombewa HDSS have been outlined elsewhere in detail [14]. In brief, verbal autopsies are conducted by qualified field workers who interview the caregivers of the deceased within approximately 1 month of death to ascertain probable causes of death [23]. Following questions regarding symptoms prior to death, the suspected cause of death is coded using the WHO-validated interVA4 software [24]. The Kombewa HDSS visits nearly 40,000 households twice yearly to measure births, deaths, and various syndromes [14]. Western Kenya has both high malaria transmission and a high prevalence of HIV [25,26]. Child mortality in the site was measured at 84.7 per 1000 live births from 2011 to 2015, with malaria and acute respiratory infections accounting for more than half of the deaths with a verbal autopsy, and diarrhea accounting for 9% of the deaths with a verbal autopsy [26]. The pneumonal conjugate vaccine (PCV10) was introduced in 2011 throughout Kenya, including the study site. Scale-up of PCV10 was rapid, with >70% of infants receiving PCV10 vaccine by 2012 [27,28]. Unfortunately, data on PCV10 coverage were not measured in the Kombewa HDSS until 2018. 

### 2.3. Variables

We considered three separate outcomes for the assessment of RV implementation: all-cause mortality among children < 3 years of age, verbal autopsy diagnosed mortality due to diarrheal disease among children < 3 years of age, and the incidence of diarrhea among children < 3 years of age. Our primary exposure was the introduction of the RV program, being split into the pre- and post-RV implementation periods, and we categorized the time as the years 2012–2014 (pre-RV) or 2015–2017 (post-RV). We repeated these analyses for infants (children < 12 months of age) with results presented as Supplemental information (Tables S1 and S2).

In addition to the outcomes and primary exposure of interest, we also included various measures hypothesized a priori to be associated with the outcomes. We considered time as monthly and included the child’s age in months as a continuous and quadratic function. In order to account for differences in household wealth, we utilized the multi-dimensional poverty index to estimate levels of deficiencies, specifically having fewer than two key assets, having a house constructed of rudimentary materials, lacking access to improved sanitation (a latrine or toilet with a smooth cleanable floor and lid to cover the hole), lacking access to an improved water source (a protected well or borehole), using a carbon-based fuel source, and not having electricity. With the exception of RV, which we measured separately, we included child vaccine status as (1) none, partial, or late (a single category) and (2) full, based on the child’s age, the recommended vaccination schedule, and the month the child received the vaccine as per the child’s vaccination card. For those children without a vaccine card whose mothers reported them to have been vaccinated, we calculated the median age at which the children received the vaccine and computed the vaccination date accordingly.

### 2.4. Data Sources and Measurement

Data were retrieved from the HDSS database on 18 June 2019. Observations were limited to the years 2012–2017. 

### 2.5. Statistical Methods

We utilized a survival analysis with a Weibull distribution to regress the exposures of interest on the mortality outcomes after accounting for various measures hypothesized a priori to be associated with child mortality. We included time, the child’s age, vaccination status, and household wealth as time-varying covariates. For the outcome of diarrhea incidence, we utilized a log-binomial model with the child as a random intercept to regress the exposures of interest on diarrhea incidence after accounting for various measures hypothesized a priori to be associated with diarrhea incidence. All analyses were performed in Stata version 15.1.

### 2.6. Ethics

Routine data collection in the HDSS was reviewed and approved by the Kenya Medical Research Institute (KEMRI) Institutional Review Board (KEMRI #1426) and the Walter Reed Army Institute of Research (WRAIR) Institutional Review Board (WRAIR #1536). Household heads provided written informed consent for themselves and other members of their household, and participation by any member was completely voluntary.

## 3. Results

### 3.1. Participants

**Figure 1:**
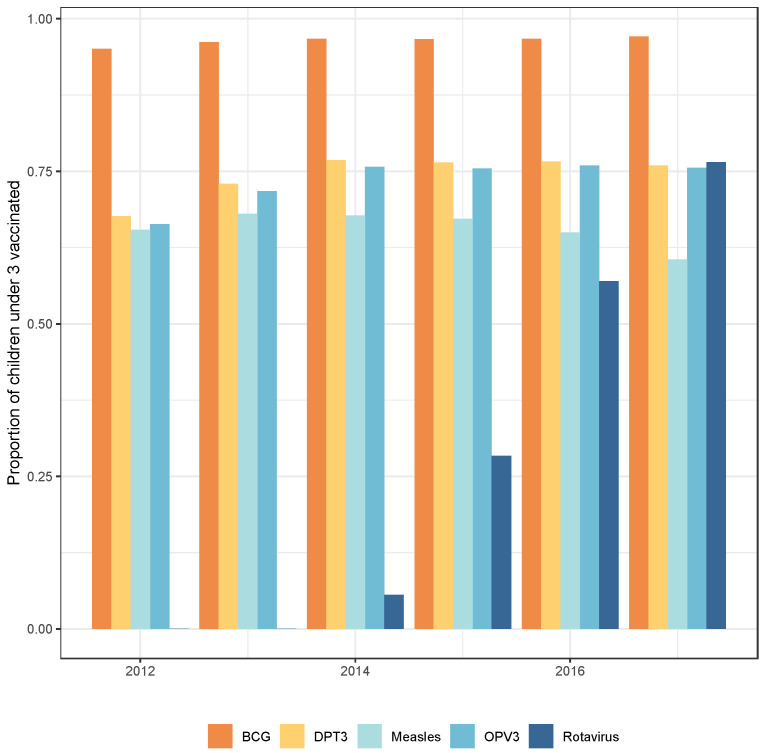
Annual vaccination coverage estimates in the Kombewa HDSS.

A total of 38,463 children under 3 years of age were included in the cohort dataset from 2012 to 2017. Of these children, a total of 26,560 (69%) could be linked with vaccination records (Table 1). Children receiving the rotavirus vaccine were more likely to be fully immunized than children not receiving the rotavirus vaccine (Table 1). Childhood vaccination remained steady throughout 2012–2017 with the exception of rotavirus, which was deployed in the study area in 2014 and increased to 75% in 2017, similar to the coverage of other childhood vaccines (Figure 1).

### 3.2. Outcome Data

**Figure 2:**
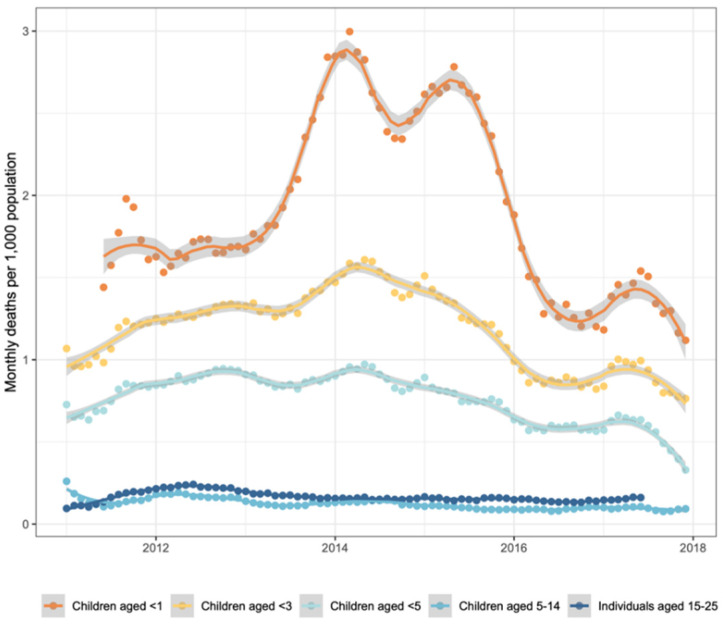
Monthly mortality trends in the Kombewa HDSS from 2012 to 2017. Monthly mortality was smoothed using locally weighted scatterplot smoothing (loess) with a span of 0.25.

A total of 937 deaths from any cause in children under 3 years of age were recorded in the HDSS dataset from 2012 to 2017 (24.4 deaths per 1000 children). Of these 937 deaths, 565 (60%) had a verbal autopsy with the primary cause of death being malaria (32%), acute respiratory infection (20%), HIV (13%), and diarrhea (10%). From the children with any form of vaccination record, a total of 618 deaths were recorded (20.0 deaths per 1000 children). Of these 618 deaths, 391 (61%) were assigned the primary cause of death as being malaria (34%), acute respiratory infections (20%), HIV (15%), or diarrhea (10%). Figure 2 shows mortality rates over time in the study period.

### 3.3. All-Cause Child Mortality

Among all children, including those without vaccination data (Table 2): From 2012 to 2014, the risk of all-cause child mortality was steady in the population (*p* = 0.159). Coinciding with the implementation of rotavirus vaccine in 2015, the risk of all-cause child mortality began to decrease by 2% each month (*p* = 0.002). Other measures such as unimproved water and sanitation, low wealth, and no electricity access were also associated with an increased risk of all-cause child mortality.

Among children with vaccination data (Table 2): From 2012 to 2014, the risk of child mortality rose by 3% each month in the population (*p* < 0.001). Coinciding with the implementation of rotavirus vaccine in 2015, the risk of all-cause child mortality decreased by 33% (*p* = 0.090), and then decreased a further 2% each month thereafter (*p* = 0.010). The receipt of the rotavirus vaccination (at least one dose) was associated with a 44% reduction in the risk of all-cause child mortality (*p* < 0.001). Other measures such as unimproved water and sanitation, low wealth, and no electricity access were also associated with an increased risk of all-cause child mortality.

### 3.4. Diarrhea-Specific Child Mortality

Among all children, including those without vaccination data (Table 3): Among all children in the HDSS, the risk of diarrhea-specific mortality was stable for the years 2012–2014. The implementation of rotavirus vaccine in 2015 was not associated with the monthly trend in diarrhea-caused mortality (*p*-value = 0.125) from 2015 to 2017. Other measures such as unimproved water and sanitation, low wealth, and no electricity access were also associated with an increased risk of diarrhea-caused child mortality.

Among children with vaccination data (Table 3): When limiting the analysis of diarrhea-specific mortality for children with vaccination records, there were only 37 diarrhea-specific deaths during the study period in the population. There was no evidence of the risk of diarrhea-caused mortality varying across time during the study period. Receiving at least one dose of the rotavirus vaccine was not associated with diarrhea-specific mortality (HR = 0.55, *p*-value = 0.331). Other measures such as unimproved water and sanitation, lower wealth, and no electricity access were also associated with an increased risk of diarrhea-caused child mortality.

### 3.5. Diarrhea Incidence

Symptoms of diarrhea in the previous two weeks were reported during nine separate rounds of HDSS data collection from 2012 to 2017. A total of 26,535 children provided data in regard to diarrhea symptoms at any time point across the time period, and 2166 (8%) reported diarrhea at any given time. The incidence of diarrhea declined 2% per month during the study period, even before rotavirus vaccine was implemented (Table 4; *p* < 0.001). The implementation of the rotavirus vaccine was not associated with diarrhea incidence across the general population without accounting for individual vaccine status (Table 4; *p* = 0.452). However, the receipt of the rotavirus vaccine was associated with a 34% reduction in the risk of diarrhea symptoms (Table 4; *p* < 0.001). Other measures such as unimproved water and sanitation were also associated with an increased risk of diarrhea symptoms.

## 4. Discussion

Our results suggest that all-cause mortality in children < 3 years declined substantially in the Kombewa HDSS, coinciding with the introduction of the RV (Table 2 and Table 3). The introduction of the RV was not associated with diarrhea-specific mortality, albeit the number of outcome was low (57 diarrhea-specific mortalities over the time period). Rotavirus vaccine implementation was not associated with the incidence of diarrhea throughout the study site (independent of rotavirus vaccine status), but those children receiving RV were much less likely to report diarrhea during follow up. These results are encouraging as they provide preliminary evidence of the population-level impact of RV in children < 3 years of age in a rural population in Kenya.

In the Kombewa HDSS, child mortality among infants and children under three years rose from 2012 to 2014, followed by a decline in 2015–2017 that coincided with RV implementation. The rise in child mortality certainly increased the likelihood of finding a downward trend with RV implementation—with any interrupted time series approach, the strength of the study design relies on the probability that the timing of the intervention aligns with a shift in the time series trend. It is likely that RV implementation contributed to but was not solely responsible for the decline in child mortality observed in 2015–2017. PCV10 was introduced in 2011 in Kenya and likely contributed to the decline in child mortality. In a similar study, King et al. attributed a similar reduction in all-cause infant mortality to PCV10 implementation; however, the authors were unable to estimate the impact of RV [29]. Furthermore we expect that rising wealth in the area likely contributed to the decline in all-cause child mortality as well [30]. 

Our study was nested in a geographically unique demographic surveillance platform, making it possible to estimate the impact of the RV program on all-cause mortality (indirect benefit), which would otherwise be difficult to estimate in facility-based studies such as those conducted in Europe [10], Latin America [8,12,13], and other African countries [6,9,31]. A unique feature of the Kombewa HDSS is the presence of a stable demography that provides reliable denominators for the assessment of trends and the impact of interventions. This is in contrast to hospital-based studies where cases are generally selected without regard to the participants’ greater population. Among those with vaccination data available, our study shows a reversal from increasing to decreasing (+3% transitioning to −2%) monthly mortality with an overall reduction of 33%, which the overall results support despite the wide confidence interval, demonstrating a positive impact on mortality attributable to the introduction of the RV. Comparable population-level-based studies in Malawi and Kenya revealed a reduction in diarrhea-specific child mortality but did not measure the impact of rotavirus vaccination on all-cause child mortality [17,31]. The reduction in all-cause child mortality post-RV implementation could be a pointer to the protection conferred by RV through the first and second years of life when most severe disease and mortality from rotavirus occur [22]. The reduction in all-cause child mortality could also be interpreted as an indirect benefit associated with RV administration (i.e., herd immunity) [32,33]. Another possibility could be whether the RV implementation campaign improved the timeliness of the administration of other vaccines, as was observed in Australia [34]. We cannot be certain with the data we have available, but we do note that children who received the RV in our cohort were more likely to be fully immunized (including at the appropriate time). 

Our study also examined changes in diarrhea-related child mortality. Among all children within the Kombewa HDSS, the risk of diarrhea-caused mortality was not associated with the introduction of the RV. The lack of association is in contrast to an observed 34% reduction in diarrhea-related child mortality post-RV implementation in a population-level study in Malawi [35]. Additionally, hospital-based studies in sub-Saharan Africa and Latin America have recorded much higher reductions in the diarrhea-related child mortality post-RV implementation of between 22 and 50% [6,9,12,36,37]. The potential explanations for this lack of association in our study could be the small numbers of reported deaths in this sub-population and incomplete verbal autopsy data. In our analysis, there were only 57 diarrhea-specific deaths during the study period, 37 of which had vaccination records. The Kombewa HDSS field procedures require the verification of vaccination records at the time of interviews. In some cases, the immunization cards were unavailable, especially when the interview was being administered to non-mothers who are the primary custodians of such information. A potential solution to the low vaccination information at the household level could be for the HDSS to collect vaccination information for all children present at the facilities and linking them back to their respective households. Such an amalgam of facility- and population-based surveillance would require a robust system of unique identification and would likely require expansion to the peripheral health facilities to capture the vaccination data maximally. A recent study in a similar HDSS setting contiguous with the Kombewa HDSS showed evidence of significant protection of up to 64% against severe rotavirus-associated hospitalization, which persisted beyond infancy [31]. However, malnutrition, which is widespread in the larger area covered by the two sites, was shown to diminish vaccine effectiveness. It is therefore probable that these factors (underweight, wasting, and stunting among children) could have played a role in limiting the vaccine protection against severe rotavirus-associated disease, dissimilar to what has been documented in Europe [10] and the United States [38]. 

Finally, we examined changes in diarrhea incidence reported during each household visit. Studies have shown that rotavirus in young children leads to transmission within household via contact [39,40,41]. We did not attempt to estimate the within-household transmission effects of a child receiving RV. However, for those children receiving RV, our results suggest that the receipt of RV is associated with a 34% reduction in the risk of diarrhea symptoms in the Kombewa HDSS population. RV has been shown to be protective of other household contacts by averting episodes or reducing symptoms among those vaccinated [42], which would potentially lead to spillover effects of the vaccine implementation. For example, a household cohort study in Malawi estimated vaccine effectiveness against rotavirus transmission at 39% (95% CI 16–57) [35]. Previous impact analyses have shown declines in rotavirus gastroenteritis hospitalizations of 43–70% globally [1,7,43,44]. In areas with a high burden of childhood diarrheal illness and death (such as our study area), a rotavirus vaccine with a 34% effectiveness can help avert more illness and death in young children. 

Our study has a number of limitations. First, children who received the rotavirus vaccine were better immunized than children who did not receive the rotavirus vaccine. It is likely that the impacts of the rotavirus vaccine that we have estimated also include some of this selection bias. Second, we were unable to include information on PCV10 vaccination status. PCV10 was introduced in Kenya in 2011 and was likely increasing in coverage throughout our study period. Unfortunately, the Kombewa HDSS did not measure PCV10 receipt until the year 2018. It is likely that the implementation of PCV10 vaccine contributed to the observed estimates of rotavirus vaccine implementation. Third, verbal autopsy has been shown to be an imprecise measurement of the cause of death [45]. However, given the limitations with vital registration systems in countries like Kenya, verbal autopsies remain important in filling the information gap in LMIC [20,23]. A large percentage of deaths in the Kombewa HDSS were not diagnosed via verbal autopsy, primarily due to challenges with funding gaps and logistics. These deaths were typically more remote from the HDSS headquarters, and less wealthy than diagnosed deaths. Nevertheless, we expect the profile of these deaths to be similar to those that were conducted via verbal autopsy, although this is a serious limitation. Fourth, the small numbers of reported deaths and variability in the percentage of deaths with verbal autopsy data and immunization data can also heavily influence results. For example, when limiting the analysis of diarrhea-specific mortality to children with vaccination records, there were only 37 diarrhea-specific deaths during the study period in the population. Another limitation is that our diarrheal deaths were non-specific for rotavirus-associated deaths. Further, we observed an incredibly stable access to sanitation over time, which could be attributable to a lack of good measures of hand hygiene or water hygiene in the data. We also consider other non-vaccine factors which can affect the observed trends over time, but there may have been other factors that were not measured that influenced our results. 

## 5. Conclusions

RV introduction in the HDSS population was followed by significant declines in all-cause child mortality and reduction in the risk of diarrhea symptoms among children younger than 3 years of age. The substantial reductions in deaths from all causes from this study illustrate the overall value of vaccination for improving child health in developing regions of the world. To maximize on the benefits associated with the vaccine, RV uptake and nutritional status need to be improved within the Kombewa HDSS population and countrywide. This analysis underscores the importance of continuous, systematic surveillance in the Kenyan population for monitoring disease burden and documenting RV vaccine impact. Our findings should encourage the consideration of rotavirus vaccine introduction in countries that have not yet introduced the vaccine, especially in rural areas with high rates of malnutrition, where vaccination may be less effective than in developed nations.

## Figures and Tables

**Table 1:** Demographic characteristics of the child cohort. Water source and sanitation access defined by the UNICEF/WHO Joint Monitoring Program. Adequate housing and assets as designated by the multidimensional poverty index. “Fully immunized” includes an appropriate timing of the vaccine as well as the dose.

		No Rotavirus Vaccine (%)	Rotavirus Vaccine (%)	No Vaccine Information (%)
Number of children		20,620 (54%)	5940 (15%)	11,903 (31%)
Deaths		519 (2.5%)	99 (1.7%)	319 (2.7%)
Sex	Female	9839 (48%)	3047 (51%)	6871 (58%)
	Male	10,781 (52%)	2893 (49%)	5032 (42%)
Water source	Improved	11,432 (55%)	3522 (59%)	7054 (59%)
	Unimproved	9188 (45%)	2418 (41%)	4849 (41%)
Sanitation access	Improved	5828 (28%)	1531 (26%)	3883 (33%)
	Unimproved	14,792 (72%)	4409 (74%)	8020 (67%)
Cooking fuel	Improved	516 (3%)	168 (3%)	371 (3%)
	Unimproved	20,104 (97%)	5772 (97%)	11,532 (97%)
Electricity	Yes	1223 (6%)	409 (7%)	932 (8%)
	None	19,397 (94%)	5531 (93%)	10,971 (92%)
Housing	Adequate	192 (1%)	82 (1%)	175 (1%)
	Inadequate	20,428 (99%)	5858 (99%)	11,728 (99%)
Assets	Adequate	13,537 (66%)	3941 (66%)	8355 (70%)
	Inadequate	7083 (34%)	1999 (34%)	3548 (30%)
Immunizations	Fully immunized	7601 (37%)	4381 (74%)	--
	Inadequately immunized	13,019 (63%)	1559 (26%)	--

**Table 2:** Survival regression results from an interrupted time series analysis of the impact of rotavirus vaccine implementation on all-cause child mortality.

		Among All Children < 3 Years of Age N = 38,463 Children; 62,856 Person Years at Risk; 937 Deaths	Among Children < 3 Years of Age with Vaccine Information N = 26,560 Children; 50,396 Person Years at Risk; 618 Deaths
Measure	Factor	Hazard Ratio (95% Confidence Interval)	Hazard Ratio (95% Confidence Interval)
Period	Pre-rotavirus vaccination	Reference	Reference
	Post-rotavirus vaccination	0.84 (0.65–1.09) *p* = 0.190	0.77 (0.57–1.04) *p* = 0.090
Post-vaccination time	Months from January 2015 (continuous)	0.98 (0.97–0.99) *p* = 0.002	0.98 (0.96–0.99) *p* = 0.010
Time	Months from January 2012 (continuous)	1.01 (1.00–1.01) *p* = 0.159	1.02 (1.02–1.04) *p* < 0.001
Child’s age	Months (continuous)	0.45 (0.41–0.50) *p* < 0.001	0.35 (0.33–0.37) *p* < 0.001
	Months (quadratic)	1.01 (1.01–1.02) *p* < 0.001	1.02 (1.02–1.02) *p* < 0.001
Water source	Improved	Reference	Reference
	Unimproved	1.20 (1.05–1.36) *p* = 0.006	1.11 (0.94–1.30) *p* = 0.214
Sanitation access	Improved	Reference	Reference
	Unimproved	1.19 (1.03–1.38) *p* = 0.019	1.36 (1.13–1.64) *p* = 0.001
Wealth (cooking fuel)	Improved cooking fuel	Reference	Reference
	Carbon-based cooking fuel	1.54 (0.87–2.73) *p* = 0.140	1.40 (0.69–2.84) *p* = 0.347
Wealth (key assets)	At least 2 key assets	Reference	Reference
	Fewer than 2 key assets	1.31 (1.14–1.49) *p* < 0.001	1.50 (1.27–1.76) *p* < 0.001
Wealth (electricity)	Electricity	Reference	Reference
	No electricity	3.03 (1.86–4.93) *p* < 0.001	2.71 (1.48–4.97) *p* = 0.001
Rotavirus immunization	None	Not included	Reference
	At least one dose	Not included	0.56 (0.43–0.74) *p* < 0.001
Immunization status	None	Not included	Reference
	Partial or late	Not included	0.51 (0.32–0.82) *p* = 0.006
	Full	Not included	0.26 (0.15–0.44) *p* < 0.001

**Table 3:** Survival regression results from interrupted time series assessing the impact of rotavirus vaccine implementation on diarrhea-specific mortality.

		Among All Children < 3 Years of Age N = 38,463 Children; 62,856 Person Years at Risk; 57 Diarrhea-Specific Deaths	Among Children with Vaccine Information < 3 Years of Age N = 26,560 Children; 50,245 Person Years at Risk; 37 Diarrhea-Specific Deaths
Measure	Factor	Hazard Ratio (95% Confidence Interval)	Hazard Ratio (95% Confidence Interval)
Period	Pre-rotavirus vaccination	Reference	Reference
	Post-rotavirus vaccination	0.68 (0.22–2.10) *p* = 0.497	0.63 (0.18–2.22) *p* = 0.473
Post-vaccination time	Months from January 2015 (continuous)	0.95 (0.88–1.02) *p* = 0.125	0.95 (0.88–1.03) *p* = 0.217
Time	Months from January 2012 (continuous)	1.00 (0.98–1.03) *p* = 0.737	1.03 (0.99–1.07) *p* = 0.110
Child’s age	Months (continuous)	0.76 (0.68–0.85) *p* < 0.001	0.80 (0.70–0.91) *p* = 0.001
Water source	Improved	Reference	Reference
	Unimproved	2.36 (1.37–4.08) *p* = 0.002	1.51 (0.79–2.89) *p* = 0.215
Sanitation access	Improved	Reference	Reference
	Unimproved	1.47 (0.79–2.74) *p* = 0.222	1.52 (0.69–3.33) *p* = 0.297)
Wealth	At least 2 key assets	Reference	Reference
	Fewer than 2 key assets	1.45 (0.85–2.46) *p* = 0.172	1.41 (0.3–2.72) *p* = 0.312
Rotavirus immunization	None	Not included	Reference
	At least one dose	Not included	0.59 (0.17–2.03) *p* = 0.401

**Table 4:** Log-binomial regression with child as a random intercept assessing the temporal trends in diarrhea incidence in regard to the implementation of the rotavirus vaccine in 2014.

		Interrupted Time Series Model (Excluding Rotavirus Vaccine Measure) N = 26,469 Children; 81,541 Observations	Rotavirus Vaccine Model (Excluding Time Components) N = 26,469 Children; 81,541 Observations
Measure	Factor	Risk Ratio (95% Confidence Interval)	Risk Ratio (95% Confidence Interval)
Post-vaccination time	Months from January 2015 (continuous)	1.00 (0.99–1.01) *p* = 0.452	Not included
Time	Months from January 2012 (continuous)	0.98 (0.98–0.99) *p* < 0.001	Not included
Rotavirus immunization	None	Not included	Reference
	At least one dose	Not included	0.66 (0.57–0.76) *p* < 0.001
Child’s age	Months (continuous)	1.18 (1.15–1.20) *p* < 0.001	1.17 (1.15–1.19) *p* < 0.001
	Months (quadratic)	0.99 (0.99–1.00) *p* < 0.001	0.99 (0.99–1.00) *p* < 0.001
Water source	Improved	Reference	Reference
	Unimproved	1.18 (1.09–1.28) *p* < 0.001	1.17 (1.08–1.27) *p* < 0.001
Sanitation access	Improved	Reference	Reference
	Unimproved	1.20 (1.09–1.31) *p* < 0.001	1.19 (1.08–1.31) *p* < 0.001
Wealth	Improved cooking fuel	Reference	Reference
	Carbon-based cooking fuel	1.17 (0.87–1.57) *p* = 0.289	1.16 (0.86–1.55) *p* = 0.289
Wealth	At least 2 key assets	Reference	Reference
	Fewer than 2 key assets	1.09 (1.00–1.19) *p* = 0.050	1.10 (1.01–1.20) *p* = 0.033
Wealth	Electricity	Reference	Reference
	No electricity	1.05 (0.87–1.27) *p* = 0.609	1.07 (0.88–1.30) *p* = 0.479

## Data Availability

The data presented in this study are available in Supplementary Materials.

## Supplementary Materials

The following supporting information can be downloaded at: https://www.mdpi.com/article/10.3390/vaccines11081299/s1, Table S1: Survival regression results from an interrupted time series analysis of the impact of rotavirus vaccine implementation on all-cause infant mortality; Table S2: Survival regression results from interrupted time series assessing the impact of rotavirus vaccine implementation on diarrhea-specific mortality.
