# Supplementary material for: Deployment of Rotavirus Vaccine in Western Kenya Coincides with a Reduction in All-Cause Child Mortality: A Retrospective Cohort Study
Source: Vaccines (Basel). 2023 Jul 29;11(8):1299. doi: 10.3390/vaccines11081299 (PMC10458991; doi:10.3390/vaccines11081299)
Supplement: Supplementary file 1 [file vaccines-11-01299-s001.zip › vaccines-2492329-supplementary.pdf]

Table S1. Survival regression results from an interrupted time series analysis of the impact of rotavirus vaccine implementation on all-cause infant mortality.

		**Among all children < 1 years of age N = 23,319 children; 18,211 person-years at risk; 359 deaths**	**Among children < 1 year of age with vaccine information N = 18,304 children; 15,046 person-years at risk; 205 deaths**
**Measure**	**Factor**	**Hazard ratio (95% confidence interval)**	**Hazard ratio (95% confidence interval)**
Period	Pre-rotavirus vaccination	Reference	Reference
	Post-rotavirus vaccination	1.20 0.81 – 1.77) p = 0.365	1.43 (0.86 – 2.39) p = 0.168
Post-vaccination time	Months from January 2015 (continuous)	0.96 (0.94 – 0.98) p = 0.001	0.94 (0.92 – 0.97) p < 0.001
Time	Months from January 2012 (continuous)	1.00 (0.99 – 1.02) p = 0.552	1.03 (1.01 – 1.05) p = 0.001
Child's age	Months (continuous)	0.01 (0.01 – 0.02) p < 0.001	0.01 (0.00 – 0.01) p < 0.001
	Months (quadratic)	1.21 (1.18 – 1.23) p < 0.001	1.29 (1.24 – 1.34) p < 0.001
Water source	Improved	Reference	Reference
	Unimproved	1.21 (0.98 – 1.49) p = 0.075	1.05 (0.80 – 1.38) p = 0.738
Sanitation access	Improved	Reference	Reference
	Unimproved	1.13 (0.90 – 1.43) p = 0.300	1.25 (0.91 – 1.72) p = 0.163
Wealth (cooking fuel)	Improved cooking fuel	Reference	Reference
	Carbon-based cooking fuel	1.14 (0.50 – 2.56) p = 0.758	0.94 (0.35 – 2.56) p = 0.905
Wealth (key assets)	At least 2 key assets	Reference	Reference
	Fewer than 2 key assets	1.18 (0.95 – 1.46) p = 0.140	1.27 (0.96 – 1.69) p = 0.099
Wealth (electricity)	Electricity	Reference	Reference
	No electricity	4.13 (1.69 – 10.10) p = 0.002	3.76 (1.18 – 11.98) p = 0.025
Rotavirus immunization	None	Not included	Reference
	At least one dose	Not included	0.41 (0.27 – 0.63) p < 0.001
Immunization status	None	Not included	Reference
	Partial or late	Not included	0.55 (0.29 – 1.02) p = 0.060
	Full	Not included	0.19 (0.09 – 0.38) p < 0.001

Table S2. Survival regression results from interrupted time series assessing the impact of rotavirus vaccine implementation on diarrhea-specific mortality.

		Among all children < 1 years of age N = 23,319 children; 19,560 person-years at risk; 33 diarrhea-specific deaths	Among children with vaccine information < 1 years of age N = 18,304 children; 15,044 person-years at risk; 21 diarrhea-specific deaths
**Measure**	**Factor**	**Hazard ratio (95% confidence interval)**	**Hazard ratio (95% confidence interval)**
Period	Pre-rotavirus vaccination	Reference	Reference
	Post-rotavirus vaccination	1.01 (0.22 – 4.62) p = 0.992	0.81 (0.14 – 4.86) p = 0.819
Post-vaccination time	Months from January 2015 (continuous)	0.95 (0.86 – 1.05) p = 0.330	0.95 (0.85 – 1.06) p = 0.320
Time	Months from January 2012 (continuous)	0.98 (0.95 – 1.02) p = 0.373	1.03 (0.98 – 1.08) p = 0.261
Child's age	Months (continuous)	0.19 (0.08 – 0.44) p < 0.001	0.08 (0.02 – 0.28) p < 0.001
Water source	Improved	Reference	Reference
	Unimproved	2.31 (1.13 – 4.70) p = 0.021	1.19 (0.50 – 2.80) p = 0.695
Sanitation access	Improved	Reference	Reference
	Unimproved	2.57 (0.99 – 6.66) p = 0.053	2.61 (0.77 – 8.89) p = 0.125
Wealth	At least 2 key assets	Reference	Reference
	Fewer than 2 key assets	0.96 (0.46 – 1.99) p = 0.916	1.01 (0.41 – 2.52) p = 0.976
Rotavirus immunization	None	Not included	Reference
	At least one dose	Not included	0.38 (0.08 – 1.81) p = 0.223
